# Effects of progressive intensity resistance training on the impact of fibromyalgia: protocol for a blinded randomized controlled trial

**DOI:** 10.1186/s12891-023-06952-3

**Published:** 2023-10-14

**Authors:** André Pontes-Silva, Almir Vieira Dibai-Filho, Thayná Soares de Melo, Leticia Menegalli Santos, Marcelo Cardoso de Souza, Josimari Melo DeSantana, Mariana Arias Avila

**Affiliations:** 1 Physical Therapy Postgraduate Program, Physical Therapy Department, Universidade Federal de São Carlos, São Carlos, SP Brazil; 2 Physical Education Postgraduate Program, Physical Education Department, Universidade Federal do Maranhão, São Luís, MA Brazil; 3 Physical Therapy Department, Universidade Federal de São Carlos, São Carlos, Brazil; 4 Postgraduate Program in Rehabilitation Sciences, Department of Physical Therapy, Universidade Federal do Rio Grande do Norte, Natal, RN Brazil; 5 Laboratory of Research on Neuroscience (LAPENE), Physical Therapy Department, Graduate Program in Health Science, Graduate Program in Physiological Science, Universidade Federal de Sergipe, Aracaju, Sergipe Brazil

**Keywords:** Fibromyalgia, Chronic Pain, Exercise, Quality of life

## Abstract

**Background:**

Fibromyalgia guidelines indicate that exercise is critical in the management of fibromyalgia, and there is evidence that patients with fibromyalgia can perform resistance training at moderate and high intensities. However, despite the biological plausibility that progression of intensity provides greater benefit to individuals, no studies have compared different intensities (progressive versus constant intensities) of the same exercise in this population.

**Objective:**

To compare the effect of 24 sessions of resistance training (progressive vs. constant intensity) on impact of fibromyalgia, sleep quality, anxiety, depression, pain, walking ability, and musculoskeletal capacity.

**Methods:**

A protocol for a blinded randomized controlled trial. The sample will be randomized into three groups: group 1 (progressive intensity, experimental), group 2 (constant intensity, control A), and group 3 (walking, control B). Group 1 will perform resistance training at moderate intensity (50% of maximum dynamic strength), previously determined by the 1 repetition maximum (1-RM) test in the proposed exercises. The strength of each individual will be reassessed every 4 weeks (by 1-RM) and the intensity of each exercise will be positively adjusted by 20% of the value observed in kg (i.e., first month 50%; second month 70%; third month 90% of the maximum dynamic strength). Group 2 will perform the same procedure, but the intensity will be maintained at 50% of the maximum dynamic strength throughout the treatment (i.e., constant intensity from the first to the third month). Group 3 will perform a 40-minute treadmill walk at low intensity, defined by a walking speed corresponding to 60-70% of the maximum heart rate, which we will control with a heart rate monitor. All groups will receive a 45-minute pain education session prior to the exercise program, covering the pathophysiologic mechanisms of chronic pain, strategies for coping with pain, avoiding hypervigilance, and deconstructing beliefs and myths about chronic pain.

**Discussion:**

The results of the present study may help health care professionals adjust the intensity of resistance training and thus plan the most effective intervention (progressive or constant intensity) to reduce the impact of fibromyalgia on patients’ lives.

**Trial registration:**

Brazilian Registry of Clinical Trials (ReBEC) ID: RBR-9pbq9fg, date of registration: October 06, 2022.

## Introduction

Fibromyalgia is a chronic condition characterized by widespread pain and complex symptoms, including fatigue, sleep disturbance, autonomic dysfunction, mood disturbance, and functional symptoms (those not explained by structural changes) [1, 2]. The prevalence varies from 2 to 6% in the world population and is more prevalent in women aged 20 to 55 years [3].

Treatment recommendations are divided into pharmacologic and nonpharmacologic [4]. Pharmacological interventions include drugs with neurological (amitriptyline, duloxetine, milnacipran, pregabalin), analgesic (tramadol), and muscle (cyclobenzaprine) effects. Nonpharmacologic interventions focus on physical therapies (acupuncture, hydrotherapy), meditative (qigong, yoga, tai chi, mindfulness), cognitive-behavioral, and physical exercise (aerobic and/or resistance training) [4].

Exercise-induced analgesia has been postulated to occur through several mechanisms: reduction of anti-inflammatory cytokines [5]; regulation of the release of some neurotransmitters that may be reduced in people with chronic pain (e.g., serotonin [5], dopamine, and norepinephrine) [6]; and cortical reorganization [7]. However, exercise-induced analgesia may not occur (or occur in a dysfunctional manner) in people with chronic pain [5, 8, 9], which may affect these patients’ adherence to treatment [9–11].

Some systematic reviews have examined different types of exercise in patients with fibromyalgia, such as flexibility [12, 13], mixed [14], resistance training [1, 2], aquatic [15], and aerobic [16]. For example, resistance training is known to be more effective than flexibility (on pain and physical function) [17], and aerobic exercise (e.g., walking) is one of the most referenced in the literature [16]. However, studies do not provide details on the appropriate procedure for adjusting the intensity of resistance training.

Evidence-based practice suggests that we should follow a tripod for health interventions: scientific evidence, professional knowledge, and patient preference [18, 19]. In this context, Andersson et al. [20] found that women with fibromyalgia prefer resistance exercises with adjusted intensity using free weights (i.e., resistance training), because the higher the load in kg (intensity) used to resist the movement, the shorter the time under muscle tension, and consequently the lower the level of stress and muscle fatigue (during and after exercise). High-intensity resistance training is also safe for people with fibromyalgia [17, 21].

However, only two systematic reviews have examined this scenario [18, 21]. The first study [17] suggests that resistance training at moderate or high intensity improves physical function in women with fibromyalgia. The second study [21] suggests the frequency (twice a week), intensity (40–80% of maximum dynamic strength), volume (1 to 2 sets of 4 to 12 repetitions of the movement), and target muscle group (gastrocnemius, quadriceps, hamstrings, pectorals, latissimus dorsi, rhomboids, deltoid, biceps, and triceps).

It is important to note that although exercise has not been shown to increase pain during its performance [22], muscle contraction can cause pain [23], and greater exercise intensity can elicit greater pain sensations, which may interfere with treatment adherence [24]. An alternative to reduce this side effect is to progressively increase exercise intensity, with loads gradually added according to biological adaptations [25]. Some studies have used this strategy in patients with fibromyalgia [22, 26–28], but the results are controversial [27–32] and the comparison between groups did not control for the progression of exercise intensity [27–32]. Studies that have used progressive intensity resistance training in patients with fibromyalgia have found reductions in disability [29] and fatigue [30].

However, the same comparison between intensities (progressive vs. constant) has not yet been performed in research on fibromyalgia, which leads to the question: does resistance training with progressive intensity, compared to constant intensity (on walking and resistance training), promote a greater reduction in the level of fibromyalgia impact? The answer to this question will characterize the scientific and social feedback of this research, which will provide subsidies to health professionals to adjust the intensity of resistance training and thus plan the most effective intervention (progressive or constant intensity; walking or resistance training) to reduce the impact of fibromyalgia in the lives of the patients.

The hypothesis is that progressive intensity resistance training in patients with fibromyalgia will produce a greater reduction in impact of fibromyalgia than constant intensity exercise (walking and resistance training). Thus, the aim of the study is to compare the effect of 24 sessions of resistance training (progressive intensity vs. constant-intensity) on impact of fibromyalgia after 24 sessions of resistance training, as well as the global perceived effect regarding treatment and impact of fibromyalgia during the intervention and after 3 months of non-exercise follow-up.

## Methods

### Trial design

This is a protocol for a blinded randomized controlled trial reported according to the Standard Protocol Items Recommendations For Interventional Trials [33]. We used the Template for Intervention Description and Replication [34] to describe the proposed intervention.

### Ethics

The research will be conducted at the Federal University of São Carlos. All procedures of this project have been approved by the Ethics Committee for Human Research of the aforementioned institution (report number: 5.499.078) and by the Brazilian Registry of Clinical Trials (ReBEC), under number RBR-9pbq9fg (available at: https://ensaiosclinicos.gov.br/rg/RBR-9pbq9fg), date of registration: October 06, 2022. We will publicize the research through social media (WhatsApp®, Facebook®, Instagram®, Twitter®) and through the University’s means of dissemination, in addition to brochures and posters in public health services in the city of São Carlos.

### Participants and settings

We will recruit individuals between the ages of 20 and 55 (which will provide greater external validity) to participate in the research through free, prior, and informed consent. Inclusion criteria: I) Diagnosis of fibromyalgia according to the recommendations of the American College of Rheumatology [35]. Non-inclusion criteria: (I) neurological conditions that would interfere with the assessments, such as paralysis, major sensory changes, and level of consciousness/understanding; (II) advanced joint disease; (III) suspected thrombosis, heart disease, and immediate postoperative period; (IV) pregnancy; (V) abuse of alcohol and illicit substances; (VI) active cancer.

### Sample size

We performed the sampling using Ene 3.0 and G*Power 3.1.9.7 software, considering the comparison of three independent groups (progressive intensity [n = 21]), group 2 (constant intensity [n = 21]), and group 3 (walking [n = 21]) at four different stages (before, during the intervention, after 24 exercise sessions, and 3 months after the end of treatment) by ANOVA for repeated measures. We chose impact of fibromyalgia as the primary outcome variable, measured by the Revised Fibromyalgia Impact Questionnaire (FIQ-R). We based the calculation on detecting the minimum clinically important difference of 27 points between independent groups (Ene) [36], standard-deviation of 16.3 points (Ene) [37], statistical power of 95% (G*Power), significance of 5% (both), effect size of 0.41 (G*Power) [38, 39], and sample loss of 15% (Ene). Thus, the sample must contain 21 individuals per group (total = 63).

### Randomization, allocation, and blinding

We will randomize the sample to assign individuals to three groups: group 1 (progressive intensity, experimental), group 2 (constant intensity, control A), and group 3 (walking, control B). The researcher responsible for recruitment, eligibility, and evaluation, as well as the statistician, will not know which group the individual is assigned to (blinding of the evaluator and statistician). The researcher responsible for administering the exercises will only open the envelopes at the time of the intervention to identify the individual and the exercise. The researcher who will have access to the final dataset of the study will not be involved in the evaluations, randomization or intervention. The database will be set up in a restricted access link and the person responsible for the data will only communicate with the evaluator.

**Fig. 1:**
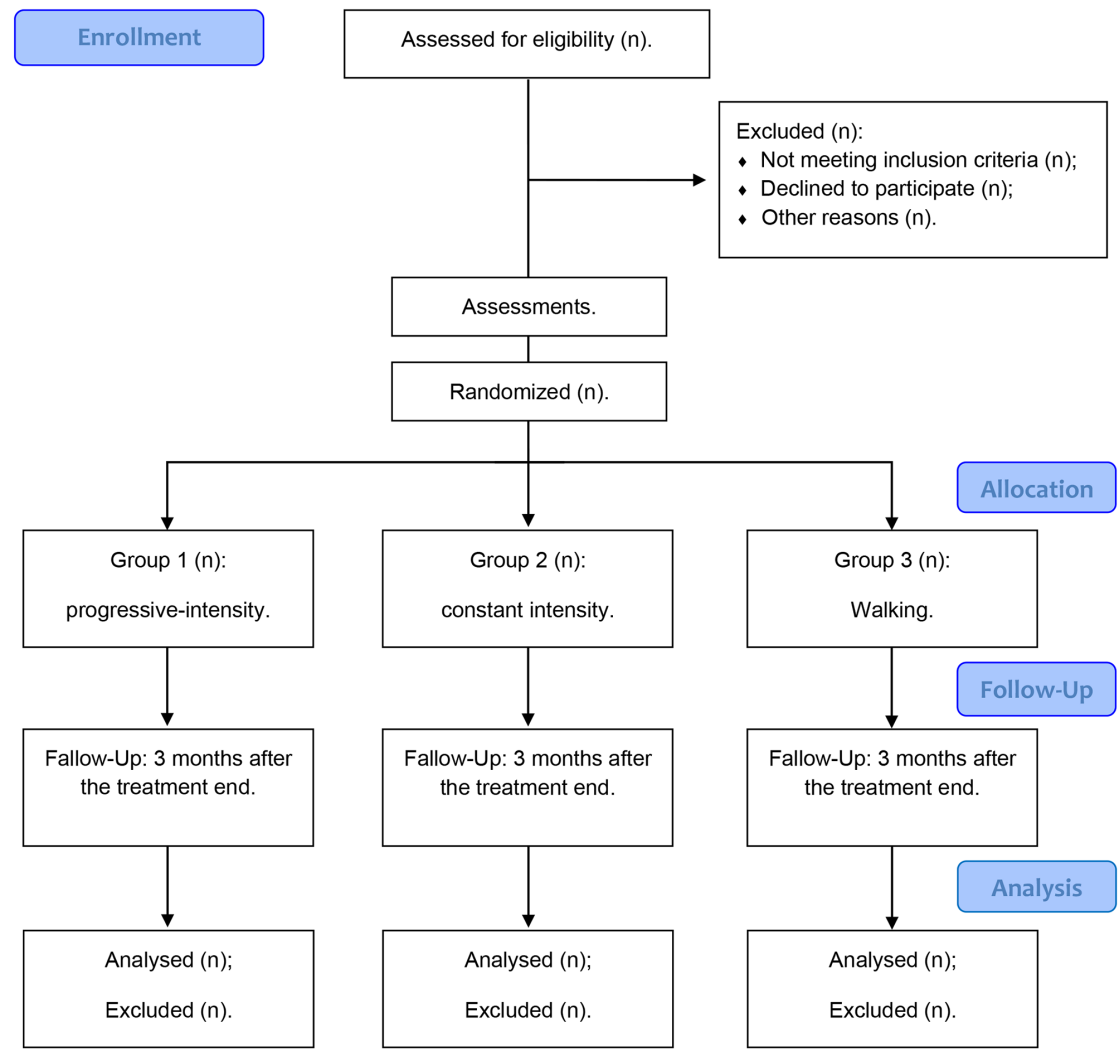
Flowchart of study

Once enrolled, baseline assessments will be conducted before participants are randomized (1:1:1) to three groups (progressive intensity, constant intensity, and walking) using simple randomization (via the website randomization.com) and allocation concealment, through opaque, sealed, and sequentially numbered envelopes, by an investigator who is not involved in the recruitment and treatment of participants (Fig. 1).

Sixty-three pieces of paper corresponding to the three groups will be placed in opaque sealed envelopes. All raters will be blinded to participant allocation. The therapist responsible for the interventions will not know the outcome of the evaluations and will only open the envelopes at the time of the intervention. Due to the proposed intervention (physical exercise), the therapist and participants will not be blinded to the intervention. Statistical analysis will be performed by a researcher blinded to the aims of the study. Participants will be instructed not to share information about the interventions with other participants and/or researchers.

### Outcomes

**Fig. 2:**
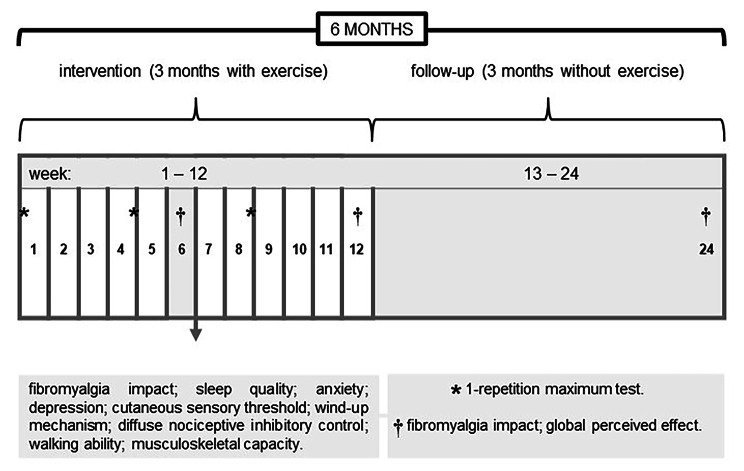
Flowchart of assessments before, during, and after 24 exercise sessions, as well as after three months of non-exercise follow-up. *Evaluation of maximal dynamic strength for exercise load, a procedure used to adjust resistance training intensity every 4 weeks (1st, 4th, and 8th week). †Assessment of the impact of fibromyalgia, as well as global perceived effect regarding treatment (6th, 12th, and 24th week)

Primary outcome will be the comparison (among groups) of the effect of 24 sessions of resistance training (progressive intensity vs. constant intensity) on impact of fibromyalgia. Secondary outcomes will be to evaluate changes in sleep quality, anxiety, depression, cutaneous sensory threshold, wind-up mechanism, diffuse nociceptive inhibitory control, walking ability, musculoskeletal capacity after 24 sessions of resistance training, as well as global perceived effect and adherence regarding treatment and impact of fibromyalgia during the intervention and after 3 months of non-exercise follow-up (Fig. 2).

### Assessments

After obtaining free, prior and informed consent, we will collect information for sample characterization. As such, initial assessment, body mass, stature, waist circumference, sex, age, comorbidities, family history, medication use, education, occupation, and impact of the Covid-19 pandemic (Table 1). In addition, we will use the instruments and tests (below) to obtain the variables mentioned in the primary and secondary outcomes.

**Table 1:** Instruments and tests to assess the participants

Outcome	Assessment (questionnaire or test)	MCID
Initial assessment	Clinical and anthropometric characteristics	n/a
Fibromyalgia screening (score)	Fibromyalgia Rapid Screening Tool [40, 41]	n/a
Impact of fibromyalgia (score)^a,b^	Revised Fibromyalgia Impact Questionnaire [42]	27
Pain level (score)^b^	Numeric Pain Rating Scale [44]	2
Sleep quality (score)^b^	Pittsburgh Sleep Quality Index [55]	n/a
Anxiety and depression (score)^b^	Hospital Anxiety and Depression Scale [57]	n/a
Cutaneous sensory threshold (score)^b^	Esthesiometry test	n/a
Wind-up (score)^b^	Temporal summation test	n/a
Diffuse noxious inhibitory controls (score)^b^	Conditioned pain modulation test	n/a
Ability to walk (m)^b^	6-Minute Walk Test	156–167
Musculoskeletal capacity (Newtons)^b^	Isokinetic dynamometer	n/a
Maximal dynamic strength for exercise load (kg)^*^	1-repetition maximum test	n/a
Global perceived effect regarding treatment (score) ^b,†^	Global Perceived Effect [65]	3

MCID: Clinically Important Minimal Difference; n/a: Not Applicable. a: Primary outcome – comparison (among groups) of the effect of 24 sessions of resistance training (progressive intensity vs. constant-intensity) on impact of fibromyalgia. b: Secondary outcome – changes after 24 sessions of resistance training, as well as the global perceived effect regarding treatment and impact of fibromyalgia during the intervention and after three months of nonexercise follow-up. *Evaluation of maximal dynamic strength for exercise load, a procedure used to adjust resistance training intensity every 4 weeks (1st, 4th, and 8th week). †Assessment of the impact of fibromyalgia, as well as global perceived effect regarding treatment (6th, 12th, and 24th week)

### Fibromyalgia screening

We will screen for fibromyalgia using the Fibromyalgia Rapid Screening Tool (FiRST) [40]. This is an instrument validated by de Sousa et al. (2022) [41] for the Brazilian population, with adequate reliability and internal consistency. It is a self-administered instrument consisting of six items with the answer options “yes” or “no”, with a cut-off score of 5 points, meaning that people who score 5 or 6 are likely to have fibromyalgia.

### Impact of fibromyalgia

We will assess the impact of fibromyalgia using the FIQ-R, an instrument validated for the Brazilian population by Lupi et al. (2016) [42], with adequate reliability and internal consistency [43]. The FIQ-R is used for assessments at week 1, 6, 12, and 24. It consists of 21 items assessing function (items 1–9), global impact (items 10–11), and symptoms (items 12–21). All questions relate to experiences during the past 7 days and are presented on an 11-point numerical rating scale (from 0 to 10). A normalization factor is applied to each of the three domain scores: the functional domain score is divided by 3, the global impact domain score is divided by 1, and the symptom domain score is divided by 2. The total FIQ-R score (from 0 to 100) is obtained by summing the three normalized domain scores. Thus, the lower the score, the lower the impact of fibromyalgia on the individual’s overall quality of life. A reduction of 27 points is considered to be the minimum clinically important difference [36], although this may change in new studies [43].

### Pain

We will assess pain with different instruments and tests. We will assess pain intensity with the Numeric Pain Rating Scale (NPRS), a self-report instrument validated in Portuguese by Ferreira-Valente et al. (2011) [44] NPRS has a sequence of numbers (from 0 to 10), where 0 represents “no pain” and 10 represents “the worst pain imaginable”. This instrument is used in tests of temporal summation (wind-up mechanism) and conditioned pain modulation (diffuse nociceptive inhibitory control). A 2-point reduction [45] in pain intensity is considered a clinically important minimal difference [46].

We will assess the cutaneous sensory threshold by means of an esthesiometry test using a set of von Frey filaments (North Coast®, Gilroy, CA, USA) in the trapezius, supraspinatus and sternocleidomastoid muscles [47]. The filaments have increasing values of compressive force (in mN), which will be tested by calibration on a precision analytical balance (CQA®, Paulínia, SP, Brazil). All subjects are blindfolded and each filament, in order of increasing force, is positioned perpendicular to the subject’s skin, gently pressed until its initial curvature, and then removed. The first filament that the individual reports having perceived touch will be considered as the cutaneous sensory threshold, so we will record the pressure force value corresponding to the reported threshold [48].

We evaluate the wind-up mechanism by means of the temporal summation test using a digital pressure algometer (ITO® brand, Tokyo, Japan), whose reliability has already been tested (Intraclass Correlation Coefficient [ICC] = 0.815) [49]. The test verifies the wind-up mechanism, which is characterized by the progressive and frequency-dependent facilitation of a neuron’s responses observed during the application of repetitive or continuous stimuli of constant intensity [50]. A pressure of 2.5 kg is applied to the anterior surface of the subject’s right forearm (7.5 cm from the distal crease of the wrist). This pressure is maintained for 30 s; during the continuous stimulus, the individual is asked about the intensity of pain felt at the 1st, 10th, 20th, and 30th second of stimulus application (using the NPRS) [51],44].

We will assess diffuse noxious inhibitory control using the conditioned pain modulation test [52], which is a phenomenon in which, under normal conditions, the perception of pain to a tested stimulus is reduced by the application of another painful stimulus (conditioned stimulus) [53]. The test will be divided into three stages: First, we will measure the pressure pain threshold on the anterior surface of the subject’s right forearm, 7.5 cm from the distal wrist crease, using a pressure algometer (ICC = 0.815) [49] to stimulate a level 4 pain (via NPRS). Second, ischemic compression (conditioned stimulus) is applied to the subject’s left arm using an analog sphygmomanometer placed 3 cm proximal to the cubital fossa. When 250 mmHg of pressure is reached, the individual is asked about the intensity using the NPRS. If the individual reports pain < 5, we will ask for flexion and extension of the elbow so that the pain increases to a level ≥ 5, and then we will reassess the pressure pain threshold on the anterior surface of the individual’s right forearm (using the pressure algometer to stimulate a level 4 pain simultaneously with the conditioned stimulus). Finally, after measuring the pressure pain threshold during the ischemic stimulus, we will remove the compression and after 30 s and then after 5 min, we will measure and record the pressure pain threshold again (using a pressure algometer to stimulate a level 4 pain on the anterior surface of the individual’s right forearm) [54].

### Sleep quality

We will assess sleep quality using the Pittsburgh Sleep Quality Index (PSQI) for sleep quality, adapted for Brazilians by Bertolazi et al. (2011) [55] It is a reliable instrument (ICC = 0.65) [56] that assesses seven sleep components: subjective quality, sleep latency, sleep duration, sleep efficiency, sleep disturbances, medication use, and daily dysfunction. For each component, the score varies from 0 to 3, and the sum gives a maximum score of 21. Scores above 5 indicate poor sleep quality.

### Anxiety and depression

We will assess anxiety and depression using the Hospital Anxiety and Depression Scale (HADS), validated for Brazilians [57]. It consists of 14 items divided into two domains (depression and anxiety) with seven items each. The items have a 4-point Likert scale (from 0 to 3), resulting in total scores ranging from 0 to 21, both for anxiety and depression. The cut off points indicating moderate to severe symptoms are: anxiety domain ≥ 8 and depression domain ≥ 9.

### Ability to walk

We will assess walking ability using the 6-minute walk test (6MWT) (CCI > 0.90) [58]. We will instruct the individual to walk at the highest possible speed during the 6 min and to stop the test if they feel uncomfortable. Before and after the session, the following variables will be monitored: systemic blood pressure, heart rate, peripheral oxygen saturation, and respiratory rate. During the session, fatigue levels (leg and respiratory) are monitored using the Borg scale. An increase of 156 to 167 m in distance walked is considered a clinically important minimum difference in individuals with fibromyalgia [59]. This test will be done before and after treatment (week 1 and week 12).

### Isokinetic dynamometry

The individuals will be positioned with the hip angle at 100º (trunk, pelvis, and thigh will be stabilized with straps). The axis of rotation of the dynamometer will be aligned with the axis of the knee, at the level of the lateral epicondyle of the femur, fixed to the distal part of the leg, approximately 5 cm above the medial malleolus. Correction for the effect of gravity will be calculated with the limb at 60° of flexion (according to the equipment instructions). We will evaluate these variables using the isokinetic dynamometer (Biodex Medical Systems 3®, Shirley, New York, USA).

A familiarization series of three submaximal isokinetic contractions is performed prior to the isokinetic assessment. 3 min after the familiarization, the subjects will perform five concentric isokinetic contractions of knee extension, from 90° to 15° (considering 0° as full extension), with a total range of motion of 75°, at an angular velocity of 60º/s (series of five repetitions), for the analysis of the variables torque peak normalized by body mass, power and work [60]. Verbal encouragement, as well as visual feedback from the equipment, will be given in an attempt to reach the maximum level of voluntary effort during all the contractions that each subject will perform. The same procedure will be repeated with the contralateral limb 5 min after the end of the dominant limb [61]. This test will be performed before and after treatment (week 1 and week 12).

### Maximal dynamic strength for exercise load

We will determine the maximum dynamic strength for exercise load using the 1 repetition maximum (1-RM) test [10]. The results of this test, in addition to being reliable (CCI > 0.90) [62], are widely used to control the intensity of resistance exercises based on the percentage of maximal dynamic strength in individuals with fibromyalgia. The individual will perform 10 repetitions of the movement (without load) for the purpose of musculoskeletal warm-up and understanding of technique. After 1 min of rest, they will perform 3 to 5 self-reported maximum repetitions, then after 3 min of rest, they will perform the 1-RM test [63].

In addition, as an alternative to confirm the test result, we will estimate the maximum dynamic strength using the mathematical equation proposed by Brzycki [64]: 1-RM = submaximal load in kg / (1.0278–0.0278 × number of repetitions). The test is used for evaluations at week 1, week 4, and week 8 to adjust the intensity of resistance training.

### Global perceived effect regarding treatment

We will assess the self-reported global perceived effect of treatment using the Global Perceived Effect (GPE) scale, an instrument validated for the Brazilian population by Costa et al. [65]. It is an 11-point descriptive scale in which the progression (or regression) of the individual’s clinical condition is classified according to their score at a given time. Thus, the individual will report their perception of improvement in the face of the intervention through a score classified as: -5 (much worse), 0 (no change), and + 5 (fully recovered). We will use the GPE at three assessments (6th, 12th, and 24th week). A change of 3 points is considered a clinically important minimum difference.

### Intervention overview

All groups will receive a 45-minute pain education session prior to the exercise program that addresses the pathophysiological mechanisms of chronic pain, strategies for coping with pain, avoiding hypervigilance, and deconstructing beliefs and myths about chronic pain (e.g., the degree of diagnostic uncertainty of the imaging exams) [66].

**Fig. 3:**
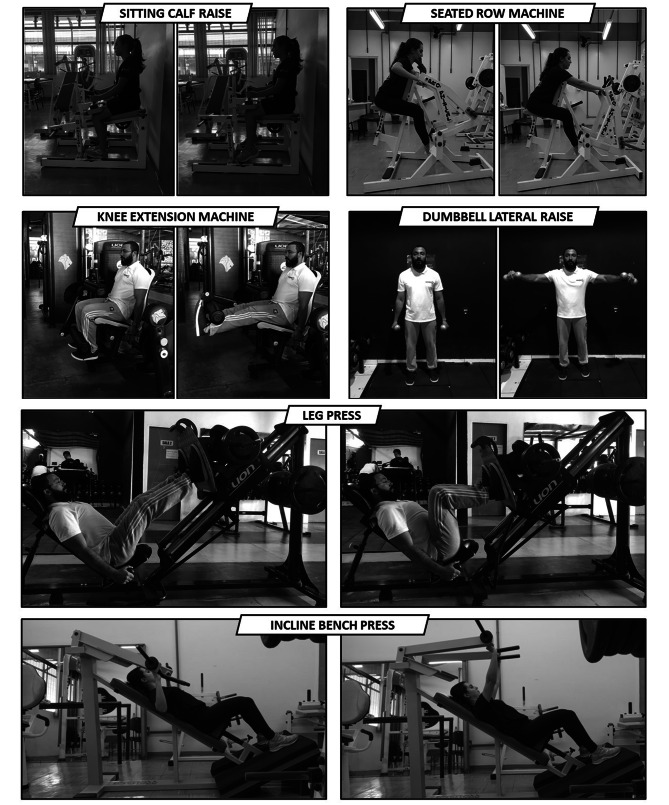
Proposed exercises

Before the first resistance training session, there will be a period of familiarization, followed by the 1-RM test in each of the exercises proposed in the resistance training program (Fig. 3). All sessions will take place individually, in a private room with lighting and air conditioning at 23 °C. All exercises will be performed by a Bachelor of Physical Education with experience in exercising individuals with chronic pain. Individuals will receive 24 exercise sessions. Two sessions per week, for 40 min and 10 min of rest after the exercise session, according to the guidelines of the American College of Sports Medicine [67], as well as clinical trials that tested this guideline in individuals with fibromyalgia [16, 17].

Each individual is instructed to discontinue the intervention at any time if they do not wish to continue with the proposed exercise session. In addition, before and after the exercise session, blood pressure (using a sphygmomanometer and stethoscope), heart rate and peripheral oxygen saturation (using an oximeter), generalized pain intensity (using the NPRS), and subjective perception (using the Borg scale) will be monitored [68].

### Group 1 (Progressive intensity, experimental)

Firstly, individuals will perform a global warm-up of four exercises (Fig. 3) without load: seated calf raise (1 min), lateral dumbbell raise (1 min), leg press (1 min), and incline bench press (1 min) [69, 70]. They will then perform moderate intensity resistance training (50% of maximum dynamic strength). Individuals work nine muscle groups (gluteus, quadriceps, hamstrings, biceps brachii, triceps brachii, pectoralis major, calf, deltoid, and latissimus dorsi) through six different exercises in the following order: seated calf raise, knee extension machine, seated row machine, dumbbell lateral raise, leg press, and incline bench press [21].

Based on 50% of the maximum dynamic strength as the initial parameter for moderate intensity in resistance training [17, 21], the load in kg will be previously identified through the 1-RM test in the proposed exercises. The individual’s muscular strength will be individually reassessed every 4 weeks (via 1-RM) and the intensity of each exercise will receive a positive adjustment of 20% of the value observed in kg (i.e., first month 50%; second month 70%; third month 90% of the maximal dynamic strength – Table 2) [17, 21].

**Table 2:** Description of the intensity of physical exercise programs

Group (intensity)	1st Month	2nd Month	3rd Month	Follow-up
1. Progressive intensity (experimental)	50% (1-RM test)	70% (1-RM test)	90% (1-RM test)	Nonexercise
2. Constant intensity (control A)	50% (1-RM test)	50% (1-RM test)	50% (1-RM test)	Nonexercise
3. Walking (control B)	60-70% (HRmax)	60-70% (HRmax)	60-70% (HRmax)	Nonexercise

1-RM test: 1-repetition maximum test; HRmax: maximum heart rate. The external load (intensity) will be adjusted on the basis of the new values observed in the 1-RM (every 4 weeks)

The intensity of the exercises (total load in kg), the interval between sets (rest in seconds), the volume (number of sets, repetitions and time under tension) as well as the frequency of the resistance training will be controlled by the researcher with experience in chronic pain. Twice a week (for 3 months), individuals with fibromyalgia will perform three sets of each exercise: 10 repetitions, 40 s of muscle tension, full range of motion, inspiration during the eccentric phase of the movement, and 60 s of rest among sets [71]. In order to maintain the same volume of exercises in resistance training (according to the intensity progression), we will add 20 s of rest to the rest among sets (i.e., first month 60; second month 80; third month 100 s of interval), because higher intensities, while maintaining the same volume of work (sets, repetitions, and time under tension), require longer intervals of rest [72].

### Group 2 (constant intensity, control A)

Firstly, individuals will perform a global warm-up of four unloaded exercises: seated calf raise (1 min), lateral dumbbell raise (1 min), leg press (1 min), and incline bench press (1 min) [69, 70]. They will then perform moderate intensity resistance training (50% of maximum dynamic strength). Individuals will work nine muscle groups (gluteus, quadriceps, hamstrings, biceps brachii, triceps brachialis, pectoralis, calf, deltoid, and latissimus dorsi) through six different exercises in the following order: seated calf raise, knee extension machine, leg press, incline bench press, seated row machine, and dumbbell lateral raise [21].

Based on 50% of the maximum dynamic strength as the initial parameter for moderate intensity in resistance training, the load in kg will be previously identified through the 1-RM test in the proposed exercises. The muscular strength of each individual will be individually reassessed every 4 weeks (via 1-RM) and the intensity (total load in kg) will be maintained at 50% of the maximum dynamic strength until the end of the complete treatment (i.e., constant intensity from month 1 to month 3 – Table 2) [17, 21].

The intensity of the exercises (total load in kg), the interval among sets (rest in seconds), the volume (number of sets, repetitions and time under tension) as well as the frequency of the resistance training will be controlled by the researcher with experience in chronic pain. Twice a week (for 3 months), individuals with fibromyalgia will perform three sets of each exercise: 10 repetitions, 40 s of muscle tension, full range of motion, inspiration during the eccentric phase of the movement, and 60 s of rest among sets. [71].

### Group 3 (walking, control B)

Individuals will walk on the treadmill for 40 min at an intensity determined by the walking speed corresponding to 60-70% of the maximum heart rate (HRmax), which will be estimated (in advance for each individual) by the mathematical equation: HRmax = 220 - age (in years) and recalculated if the individual has a birthday during the treatment period [67]. As such, the individual is verbally motivated to walk at a constant pace to keep the heart rate around 60-70% of HRmax (Table 2).

To ensure stabilization of low intensity, if the individual exceeds 60–70% of HRmax, we slowly reduce the treadmill speed until the heartbeats reach 60-70% of HRmax (considering the standard error of estimation of up to 10 heartbeats for more or less) [67]. Heart rate is monitored using a Polar V800 heart rate monitor (Polar Electro OU®, Kempele, Finland) with a sensor attached to the subject’s chest. This device has been used in studies of chronic pain [73, 74].

### Statistical analysis

We will perform statistical analysis based on intention-to-treat analysis. We will use histograms and normality tests to verify the distribution of the data. Comparisons will be made through mixed linear models using interaction between factors time (before, during the intervention, after 24 exercise sessions, and 3 months after the end of treatment) and group (progressive intensity, constant intensity, and walking), besides, the baseline will be used as a covariate in the analyses [75]. Data will be presented as mean, standard-deviation, difference between means, confidence interval (95%) of these differences, and effect size (Cohen d). We will consider a significance level of 5% on the SPSS® software, version 17.0 (Chicago, IL, EUA) [76].

## Discussion

### Potential impact and significance of the study

Exercise science has shown that progressive intensity resistance training generates better musculoskeletal adaptations than constant-intensity resistance training [77, 78]. These adaptations contribute positively to the physical function [13, 17], cognitive performance [4, 79], and patient quality of life [4]. However, these interventions [77, 78] and outcomes [4] have not yet been studied in patients with fibromyalgia [13, 17].

To our knowledge, this study will be the first clinical trial to compare resistance training intensity (progressive vs. constant) in patients with fibromyalgia. Therefore, the results will show the effectiveness (or not) of this proposal. In addition, as a randomized clinical trial, it will contribute to novel evidence syntheses in systematic reviews [17], as well as guidelines [4], generating more perspectives for evidence-based health [18, 19]. Besides, this study compares progressive intensity to two types of exercise (resistance and aerobic), thus filling two gaps simultaneously.

### Contribution to professionals and patients

This study will provide health professionals with guidance in planning/applying resistance training [17] (with progressive intensity [77] or not) to reduce the impact of fibromyalgia [42], as we will find out if gradual exposure to resistance training [17] is more effective than constant exposure (to resistance training and/or walking) on patients’ rehabilitation. In addition, the results will also contribute to patients’ knowledge about non-pharmacological treatment of fibromyalgia [4] and whether the proposed types of exercise are significantly different on the primary outcome (impact of fibromyalgia [42]). In fact, if the results show insignificant differences [76] among the proposed exercises, patients with fibromyalgia will be able to choose the type and intensity of exercise according to their preferences [19, 20].

### Strengths and weaknesses of the study

The strength of this study will be the comparison of the progressive intensity of resistance training with the constant intensity of two types of exercise (resistance training and walking). In contrast, the weakness of this research will be the impossibility of blinding the therapist and the patient during the treatment, considering that the intervention type (physical exercise) will be observed by both.

### Prospects for future research

Although this study focuses on the investigation of exercise intensity on the impact of fibromyalgia [42], there is still a need for future studies to propose ways to compare different types of exercise (e.g., resistance training vs. walking), as we know that musculoskeletal adaptations result from the physical effort performed (not the type of exercise proposed) [71]. This means that further studies investigating different exercise types should develop strategies or methods to monitor the total physical effort performed in each exercise program [72].

## Data Availability

The data and materials in this paper are available from the corresponding author on request.

## Abbreviations

1-RM: 1-repetition maximum
6MWT: 6-Minute Walk Test
FIQ-R: Revised Fibromyalgia Impact Questionnaire
FiRST: Fibromyalgia Rapid Screening Tool
GPE: Global Perceived Effect
HADS: Hospital Anxiety and Depression Scale
HRmax: maximum heart rate
ICC: Intraclass Correlation Coefficient
NPRS: Numeric Pain Rating Scale
PSQI: Pittsburgh Sleep Quality Index
ReBEC: Brazilian Registry of Clinical Trials
